# Perceived impacts of extreme heat on health and livelihoods in Nigeria

**DOI:** 10.1088/2752-5309/ae73a7

**Published:** 2026-06-11

**Authors:** Adetoun Mustapha Olaitan, Ifeoma Egbogah, Kazeem Adewale Osuolale, Chinaza Esiaba, Lisa Reyes Mason, Christine C Ekenga

**Affiliations:** 1 Lead City University, Ibadan, Nigeria; 2 Nigerian Institute of Medical Research, Edmund Crescent, Yaba, Lagos, Nigeria; 3 Environmental Quality for Public Good, Lagos, Lagos State, Nigeria; 4 Gangarosa Department of Environmental Health, Rollins School of Public Health, Emory University, Atlanta, GA, United States of America; 5 Graduate School of Social Work, University of Denver, Denver, CO, United States of America

**Keywords:** extreme heat, Nigeria, climate, public health

## Abstract

Extreme weather events are becoming more frequent and severe in Nigeria, yet empirical evidence on their health and livelihood impacts, particularly across gender and socioeconomic lines, remains limited. This study examines how Nigerian adults perceive and are affected by extreme heat during the dry season, focusing on physical, mental, and financial impacts. A cross-sectional survey was conducted among 368 adults, capturing sociodemographic information, frequency of exposure to hot temperatures and drought, perceived health and financial impacts, and coping strategies. Bayesian cumulative logit models were used to assess associations between heat exposure and adverse outcomes, controlling for gender, income, social cohesion, and region. The sample included 217 men and 151 women, who are mostly educated professionals in urban settings. A substantial proportion of participants reported that extreme heat ‘always’ or ‘usually’ affected their physical health (33% of men, 26% of women), mental health (20% of men, 11% of women), and financial well-being (16% of men, 9% of women). Most coping strategies were individual and low-cost, such as taking more showers or drinking more water, while community-oriented behaviors were rare. In multivariable models, perceived frequency of heat was not strongly associated with adverse impacts, but greater perceived drought was significantly linked to higher odds of financial hardship (OR: 1.76, 95% CI: 1.07–2.93). Women reported lower odds than men of experiencing greater physical health impacts (OR: 0.58, 95% CI: 0.33–0.98). These findings highlight that extreme heat is a significant multisystem stressor in Nigeria, with drought more consistently driving financial strain. The predominance of individual coping strategies suggests a need for integrated interventions that combine infrastructure improvements with community-based resilience efforts to mitigate the multifaceted impacts of extreme heat. Observed gender differences in physical health impacts indicate that adaptation strategies should be gender-responsive and sensitive to local labor patterns, mobility and access to resources.

## 1. Introduction

### 1.1. Extreme weather events (EWEs) and climate change

EWEs, including floods, heatwaves, wildfires, storms, droughts, and high winds, are intensifying as climate change progresses globally. In West Africa, and especially Nigeria, these disturbances are a direct consequence of anthropogenic climate change, resulting in shifts to regional weather patterns defined by heightened frequency and severity of EWEs [1, 2]. Illustrative events such as the August 2024 flooding in Borno State—triggered by structural damage to the Alau Dam and torrential rainfall—highlight the scale of human and infrastructure impacts, with over 239 000 people in Maiduguri Metropolitan Council and Jere Local Government Areas affected [3]. Neighboring Adamawa and Yobe States also experienced significant disruption, while anticipated water releases from Cameroon’s Lagdo Dam raise ongoing concerns for further threats [3].

The Intergovernmental Panel on Climate Change defines weather extremes as events distinguished by their abnormal intensity or infrequency when compared to historical patterns [4]. Nigeria’s coastal location exposes the country to a spectrum of climate hazards, making it one of the world’s most vulnerable regions, with roughly 6% of its land area at high risk of climate-induced events [5]. In addition to the direct impact of flooding, droughts reduce water availability and agricultural productivity, compounding food insecurity, while heatwaves trigger acute health stress, especially in urban areas with limited green spaces.

Limited vegetation and tree cover reduce natural cooling processes such as shading and evapotranspiration, while extensive paved and built surfaces absorb and retain heat during the day and release it slowly at night [6]. This process contributes to the urban heat island effect, where temperatures in densely built urban areas can be significantly higher than in surrounding rural areas, thereby increasing residents’ exposure to extreme heat and associated health risks [6].

### 1.2. Climate vulnerability and socioeconomic dynamics in Nigeria

Nigeria’s vulnerability to climate change extends far beyond physical environmental factors, as socioeconomic conditions intensify its impacts. Across the country, inadequate infrastructure, limited healthcare access, and low adaptive capacity amplify the health and livelihood consequences of EWEs [7]. These challenges require region-specific assessments rather than global generalizations. Northern Nigeria exemplifies this vulnerability, experiencing acute climate shifts that disproportionately affect land-dependent populations [8]. Drought and rising temperatures threaten natural resources, particularly water and arable land, placing immense strain on livelihoods. As agricultural yields decline and land becomes less productive, displacement, social unrest, and criminal activity often increase [9]. In turn, the disruption of land-based occupations can undermine both household economies and broader community stability, weakening social cohesion and residents’ sense of belonging. Evidence also links climate-related economic pressures to rising domestic and community violence [10]. Yet, many communities also display resilience, reporting post-disaster growth, altruism, and compassion alongside experiences of distress, anxiety, and fear [11].

Among all EWEs, heatwaves and droughts impose the most distinctive and compounding burdens. In contrast to sudden disasters such as floods, these are slow-onset events whose cumulative effects often escape immediate attention. Prolonged heat and water scarcity damage agricultural productivity and threaten food security and livelihood [12]. These conditions also deepen existing gender inequalities: women and girls, who frequently bear responsibility for water collection and household food, face increased exposure to both environmental and social risks [13, 14]. Longer search times for water and fuel increase their physical and emotional stress and vulnerability to violence. Hence, addressing climate vulnerability in Nigeria demands integrated responses attentive to socioeconomic inequities and gendered dimensions of environmental stress.

However, the extent to which these gendered vulnerabilities translate into differential health and livelihood impacts during extreme heat remains context-specific and may depend on labor roles, mobility patterns, housing conditions and access to resources.

### 1.3. Addressing evidence gaps in West Africa

Despite the mounting evidence of climate change impacting health and economies globally, there remains a significant gap of region-specific research focusing explicitly on the compounded effects of extreme heat in West Africa [15, 16]. Most systematic reviews and meta-analyses concentrate on other regions. This geographic disparity highlights significant gaps in data availability, quality, and contextual relevance for West African populations where vulnerabilities are heightened and resources scarcer [16].

The compounded effects of extreme heat necessitate an integrated assessment framework that simultaneously considers physical health, psychological distress, and financial strain to effectively guide intervention and policy. The lack of standardized health assessment tools and economic impact metrics tailored to West African contexts further hampers accurate evaluation and resource allocation. Given the region’s demographic characteristics, dependency on climate-sensitive livelihood sectors like agriculture, and fragile health infrastructures, there is a pressing need to generate localized evidence on the impacts of thermal stress and water scarcity.

To help address this gap, this study presents an exploratory cross-sectional investigation of the perceived physical, mental, and financial impacts of extreme heat among adults in Nigeria. The study focuses on individuals’ perceptions of heat and drought conditions and the ways in which these conditions affect their health and livelihoods. By examining these interconnected dimensions and exploring potential gender and sociodemographic differences, the study provides preliminary insights into how people experience and respond to extreme heat during the dry season. As an exploratory study, the findings are intended to contribute first knowledge that can inform future research and more comprehensive assessments of climate-related health and livelihood risks.

This study therefore addresses the following research questions:

1. What is the perceived prevalence of physical, mental, and financial impacts associated with very hot temperatures during the dry season among adults in Nigeria?
2. What coping behaviors do individuals report adopting during periods of extreme heat?
3. Are perceived frequencies of extreme heat and drought associated with perceived physical, mental, and financial impacts after accounting for key sociodemographic factors?
4. Are there gender differences in perceived health and financial impacts associated with extreme heat and drought?

## 2. Methods

### 2.1. Survey sample and administration

We conducted a cross-sectional survey between May 2021 and April 2022 to assess the physical, mental, and financial health impacts of EWEs in Nigeria. Data were collected using a previously standardized questionnaire that was adapted to the Nigerian context. The questionnaire contained items on demographic and socioeconomic characteristics, experiences with weather extremes during both the dry and rainy seasons, perceived health and financial impacts, adaptive strategies, preparedness, and community resilience. Extreme heat-specific items addressed exposure to heat and drought, associated health outcomes, financial disruptions, and coping behaviors and strategies. The survey was administered either online or in printed self-administered formats, depending on participants’ access and preference. Due to funding constraints, interviewer-administered questionnaires were not feasible.

The study sample comprised adults residing in Nigeria. Eligible participants were:

1. Aged 18 years or older, and
2. Able to read, speak, and understand English.

The English-language inclusion criterion was necessary because the questionnaire was available only in English. The implications of this limitation, including the exclusion of non-English literate populations, will be considered in the discussion. This study received ethical approval from the Institutional Review Boards of the Nigerian Institute of Medical Research (IRB/21/039) and Washington University in St. Louis (202105066). The research was conducted in accordance with the principles embodied in the Declaration of Helsinki and in accordance with local statutory requirements. All participants gave written informed consent to participate in the study.

### 2.2. Sampling strategy and sample size determination

A convenience sampling approach was used to recruit participants from various states across Nigeria. Recruitment was carried out through both online dissemination (via email lists, social media, and professional networks) and physical distribution of printed surveys in selected communities. Informed consent was obtained from all study participants.

This study was designed to generate preliminary evidence on the health impacts of EWEs in Nigeria [17]. Sample size and power calculations were conducted using SAS simulation code provided by Dang *et al* [18] to determine the minimum number of participants needed to detect meaningful associations across outcome domains.

All calculations assumed a two-sided test, an alpha level of 0.05, and 80% power.

• For health impacts, a sample size of 136 participants was required to detect an effect size of 0.68.
• For financial impacts, a sample size of 118 participants was required to detect an effect size of 0.54.

To account for potential nonresponse, a minimum recruitment target of 140 participants was set for the study.

### 2.3. Measures

#### 2.3.1. Extreme heat exposures

To assess frequency of exposure to extreme heat, participants were asked, ‘How often do very hot temperatures occur during the dry season?’ Response options included: ‘Every day’, ‘A few times a week’, ‘Once per week’, ‘A couple of times per month’, ‘Once per month’, ‘Less than once a month’, or ‘Never.’ For statistical analyses, responses were recoded into two categories: ‘Frequently’ (combining ‘Everyday’ and ‘A few times a week’) and ‘Rarely/Occasionally’ (combining ‘A couple of times per month’, ‘Once per month’, ‘Less than once a month’, and ‘Never’).

#### 2.3.2. Health and livelihood impacts

The endogenous variables in this study include mental health impacts, physical health impacts, and financial impacts. To assess mental and physical health outcomes, participants were asked: ‘To what extent is your [mental/physical] health negatively affected by very hot temperatures during the dry season?’ Financial impact was measured using two questions: ‘Is your financial health negatively affected by very hot temperatures during the dry season?’, ‘Are your finances negatively affected by drought during the dry season?’ For each item, participants responded using a five-point scale: Always, Usually, Sometimes, Rarely, and Not at all. During analysis, the categories ‘Always’ and ‘Usually’ were combined, as were ‘Sometimes’ and ‘Rarely.’ This recoding was necessary to address issues of sparse data and problematic separation resulting from small response frequencies in some categories.

#### 2.3.3. Coping behaviors

Coping behaviors were assessed with a multiple-response survey item. Participants were asked, ‘When the temperature is very hot during the dry season, which of the following do you do?’ Response options included closing curtains or shades, opening windows, taking more showers or baths, using fans at home, using air conditioning at home, drinking more water or cold drinks, changing their diet, checking on neighbors to see if they are safe, staying outside under shade, and going someplace other than their home to stay cool.

#### 2.3.4. Demographic and contextual measures

The demographic variables in this study included age (years), marital status (married, separated or divorced, single, and widowed), income (low income <₦299 999), medium income (₦300 000–₦2999 999), and high income (>₦3000 000)), education level (post-secondary, secondary, learned a trade, primary education and no formal education) and occupation (professional or non-professional).

Contextual measures included neighborhood social cohesion and regional zone of residence. Neighborhood social cohesion was assessed using the *Social Cohesion and Trust* scale, which includes five items such as ‘People in my neighborhood are willing to help their neighbors.’ Each item was rated on a five-point Likert scale from 1 = strongly disagree to 5 = strongly agree. Scores were averaged to obtain a composite measure, with items reverse-coded where necessary. For administrative planning purposes, Nigeria is usually divided into six geopolitical zones. We coded participants’ zone of residence according to their state’s geopolitical zone: North East (Adamawa, Bauchi, Borno, Gombe, Taraba, Yobe), North Central (Benue, Kogi, Kwara, Nasarawa, Niger, Plateau, Federal Capital Territory [FCT] Abuja), North West (Jigawa, Kaduna, Kano, Katsina, Kebbi, Sokoto, Zamfara), South East (Abia, Anambra, Ebonyi, Enugu, Imo), South South (Akwa Ibom, Bayelsa, Cross River, Delta, Edo, Rivers), and South West (Ekiti, Lagos, Ogun, Ondo, Osun, Oyo).

### 2.4. Statistical analyses

A mixed analytic approach was employed. First, descriptive and exploratory analyses were conducted to summarize sample characteristics and assess the distribution of study variables. Next, Kendall’s tau-b correlations were used to examine bivariate associations between self-reported exposures (heat and drought) and outcomes (physical, mental, and financial impacts). Finally, Bayesian cumulative logit models with partial proportional odds specifications were fitted to identify multivariable predictors of the ordered outcomes (‘Not at all,’ ‘Sometimes,’ ‘Always’). Weakly informative priors were applied, and sparse response categories were collapsed to improve model stability. Predictors included frequency of exposure to heat and drought, as well as gender, income, neighborhood cohesion, and zone of residence while age and occupation were included as covariates. Models were estimated using the brms package in R (version 4.4.2), and model convergence was verified through $\hat{R}$ statistics and effective sample sizes.

## 3. Results

### 3.1. Sample characteristics

Table 1 displays the characteristics of the study sample by sex. Among the 368 participants, 151 were female and 217 were male. Women were slightly younger than men (mean age 32 years, SD 8 vs 36 years, SD 9). Most participants were married (57% of women and 58% of men). Men were more likely to report high income (13% vs 8.0% among women), whereas medium income was the most common category for both sexes (51% of women and 55% of men). Educational attainment was high in both groups, with 86% of women and 85% of men reporting postsecondary education. Men were more often employed in professional occupations (59% vs 50% among women), while unemployment or nonreporting of occupation was common in both groups (30% of women and 22% of men). Neighborhood social cohesion scores were similar for women and men. Participants resided in all six geopolitical zones of Nigeria (see appendix figure), although most lived in the South West (71% of women and 58% of men).

**Table 1.** Characteristics of the study sample (*n* = 368)*.

	Female *n* = 151	Male *n* = 217
Age		
Mean (SD)	32 (8)	36 (9)
Median (Q1, Q3)	31 (27, 36)	34 (30, 40)
Min, max	18, 65	18, 67

Marital status *n* (%)		
Married	86 (57%)	125 (58%)
Separated or divorced	1 (0.7%)	0 (0%)
Single	63 (42%)	91 (42%)
Widowed	1 (0.7%)	0 (0%)

Income *n* (%)		
High	11 (8.0%)	28 (13%)
Medium	71 (51%)	117 (55%)
Low	56 (41%)	66 (31%)

Education *n* (%)		
Post secondary	128 (86%)	185 (85%)
Secondary	16 (11%)	27 (12%)
Primary	1 (0.7%)	4 (1.8%)
Learned trade	2 (1.4%)	1 (0.5%)
No education	1 (0.7%)	0 (0%)

Occupation *n* (%)		
Professional	76 (50%)	129 (59%)
Non-professional	29 (19%)	40 (18%)
Unemployed/not reported	46 (30%)	48 (22%)

Neighborhood social cohesion *n* (%)		
Mean	3.48 (0.54)	3.51 (0.55)
Median (Q1, Q3)	3.60 (3.20, 3.80)	3.60 (3.20, 3.80)
Min, max	2.00, 5.00	1.40, 5.00

Zone of residence		
North Central	15 (10%)	35 (17%)
North East	3 (2.0%)	5 (2.4%)
North West	4 (2.7%)	29 (14%)
South East	6 (4.0%)	7 (3.3%)
South South	15 (10%)	13 (6.1%)
South West	106 (71%)	123 (58%)

The following variables are missing data for a subset of participants: marital status (*n* = 1), income (*n* = 19), education (*n* = 3), neighborhood social cohesion (*n* = 8); zone (*n* = 7).

### 3.2. Health and livelihood impacts

**Figure 1.**
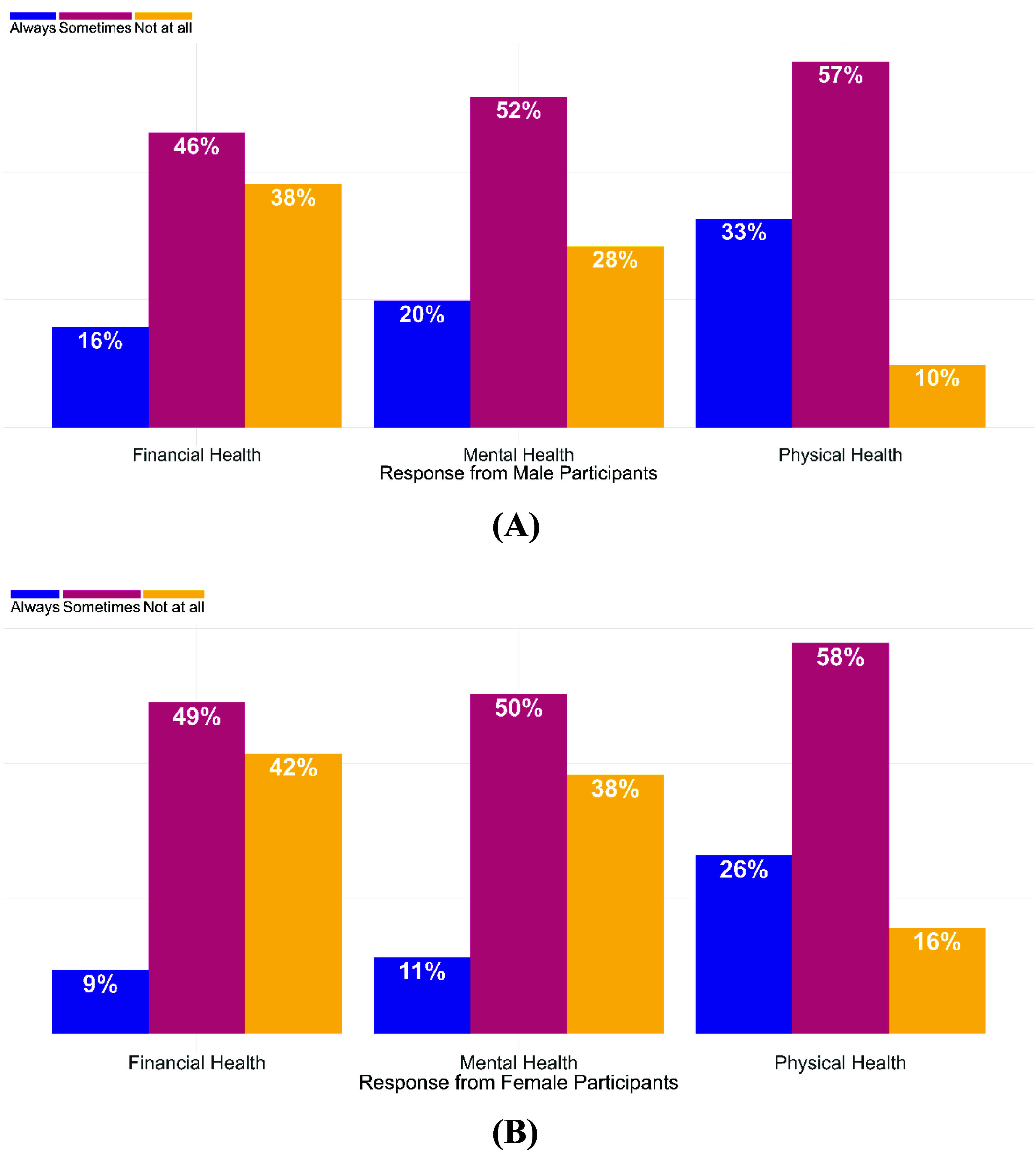
(A) Health and livelihood impacts of heat among male participants. (B) Health and livelihood impacts of heat among female participants.

Figure 1 presents the distribution of self-reported mental, physical, and financial impacts of heat among male (figure 1(A)) and female (figure 1(B)) participants. Overall, men reported that very hot temperatures during the dry season ‘always’ or ‘usually’ affected their physical (33% men and 26% women, respectively), mental (20% men, 11% women) and financial (16% men, 9% women) health at higher rates than women.

### 3.3. Coping behaviors

**Figure 2.**
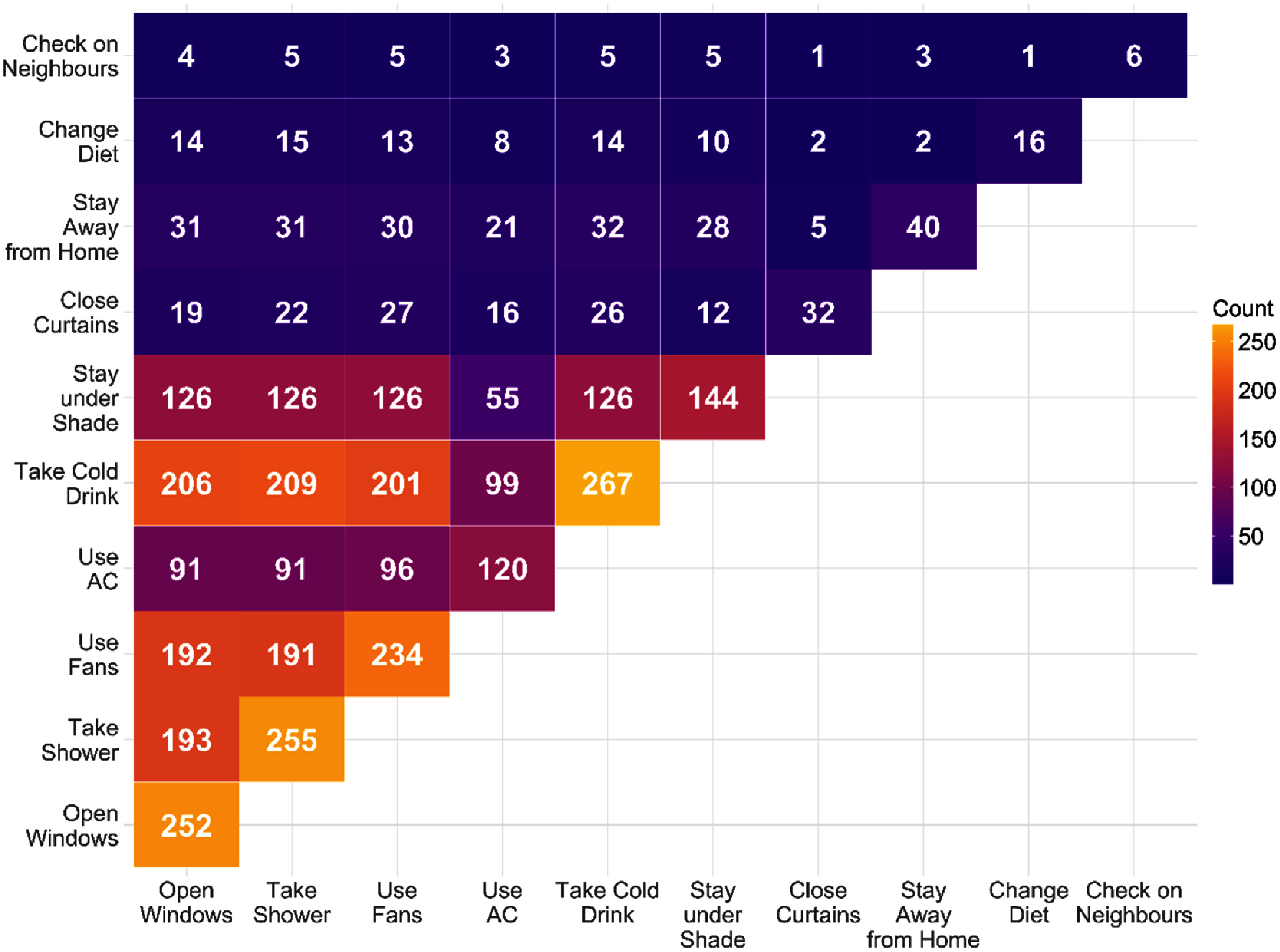
Co-occurrence of coping behaviors during dry season conditions.

Figure 2 illustrates the co-occurrence of coping behaviors that participants reported using during very hot dry-season conditions. Participants most commonly combined indoor cooling behaviors with drinking more water or cold drinks. Taking more showers or baths at home and drinking more water or cold drinks was the most frequent combination (57%), followed by opening windows and drinking more water or cold drinks (56%), and using fans and drinking more water or cold drinks (55%). Less common were combinations that involved changing diets (4%) or checking on neighbors (1%).

### 3.4. Correlations between heat, drought, and adverse health and financial impacts

Table 2 presents Kendall’s rank correlation coefficients assessing the relationships between two climate-related exposures (heat and drought) and self-reported physical, mental, and financial impacts during the dry season. Kendall’s rank correlation analyses showed that more frequent exposure to very hot temperatures during the dry season was modestly associated with greater self-reported physical health impacts (Ꞇ = 0.109, *p* = 0.018). In contrast, heat exposure was not significantly associated with self-reported mental health or financial impacts (Ꞇ = 0.025, *p* = 0.580) or financial impacts (Ꞇ = − 0.016, *p* = 0.729), suggesting that higher temperatures alone may not directly influence mental well-being or financial strain in this population. By comparison, more frequent drought was correlated with greater perceived financial strain (Ꞇ = 0.156, *p* < 0.001).

**Table 2.** Kendall’s rank correlations between heat and drought exposures and self-reported physical, mental, and financial impacts during dry season.

Exposure	Impact	Kendall’s Ꞇ	*P*-value
Heat	Physical health	0.109	0.018^*^
	Mental health	0.025	0.580

	Financial	−0.016	0.729
Drought	Financial	0.156	<0.001^*^^*^^*^

^*^ *p* < 0.05; **p < 0.01; ***p < 0.001.

### 3.5. Factors associated with adverse heat and financial impacts

In multivariable Bayesian cumulative logit models, more frequent exposure to very hot temperatures during the dry season was not associated with higher odds of reporting physical, mental, or financial impacts (table 3). Women had lower odds than men of reporting greater physical health impacts (OR: 0.58, 95% CI: 0.33–0.98), whereas associations between gender and mental or financial impacts were attenuated, but not significant. Income, neighborhood social cohesion, and geopolitical zone showed no strong evidence of association with any outcome. Higher perceived drought during the dry season was associated with greater odds of reporting financial impacts (OR: 1.76, 95% CI: 1.07–2.93).

**Table 3.** Associations between heat and drought exposures and self-reported physical, mental, and financial health impacts during dry season.

Predictor	Physical OR (95% CI)	Mental OR (95% CI)	Financial OR (95% CI)
Heat	1.17 (0.79–1.74)	0.96 (0.64–1.43)	1.05 (0.72–1.55)

Gender			
Female	0.58 (0.33–0.98)	0.70 (0.42–1.18)	0.80 (0.48–1.33)
Male	Reference 1.0	Reference 1.0	Reference 1.0

Income			
Medium income—Tier 1	0.74 (0.31–1.70)	1.00 (0.55–1.79)	0.75 (0.42–1.35)
Medium income—Tier 2	0.93 (0.51–1.71)	1.16 (0.58–2.42)	1.03 (0.53–2.01)
High income—Tier 1	0.70 (0.23–2.22)	1.56 (0.65–4.14)	0.92 (0.40–2.13)
High income—Tier 2	1.26 (0.54–2.91)	0.66 (0.22–1.83)	0.50 (0.17–1.35)
Low income	Reference 1.0	Reference 1.0	Reference 1.0

Neighborhood social cohesion	0.92 (0.65–1.29)	0.86 (0.61–1.21)	0.79 (0.55–1.11)
Zone			
North East	1.14 (0.32–3.88)	0.65 (0.20–2.17)	1.70 (0.50–5.86)
North West	1.50 (0.60–3.79)	1.55 (0.59–4.09)	1.64 (0.67–3.97)
South East	2.92 (0.91–9.88)	1.46 (0.46–4.63)	0.35 (0.11–1.12)
South West	0.92 (0.50–1.74)	0.80 (0.43–1.49)	0.58 (0.31–1.09)
South South	1.02 (0.39–2.72)	0.41 (0.15–1.12)	0.88 (0.33–2.26)
North Central	Reference 1.0	Reference 1.0	Reference 1.0

Drought^a^			1.76 (1.07–2.93)

^a^ The drought variable assessed financial impacts only, not physical or mental health impacts.

## 4. Discussion

This exploratory study examined self-reported impacts of extreme heat and drought on physical health, mental health and financial wellbeing among adults in Nigeria. A substantial proportion of respondents reported that very hot temperatures during the dry season negatively affected multiple aspects of wellbeing, and coping strategies were dominated by low-cost, individual behaviors such as increased water consumption, bathing and use of fans.

### 4.1. Key findings and interpretations

A large majority of participants reported that very hot dry season temperatures affected their physical and mental health, and approximately 40% reported financial impacts. These prevalences indicate that extreme heat is a regular, everyday multisystem stressor during dry season. Although frequent heat exposure was positively associated with adverse physical health impacts in the study, consistent with previous studies that identifies heat as a public health risk in sub-Saharan Africa [19], this association was not statistically significant after adjusting for other variables in the multivariable analysis. Additionally, no meaningful associations were found between heat exposure and either mental health or financial impacts.

Several explanations may account for this pattern. First, the exposure variable captured perceived frequency of hot temperatures rather than individual-level heat exposure, which may vary substantially depending on occupation, housing characteristics, daily activities, and access to cooling resources. Second, limited variability in perceived exposure or potential measurement error may have reduced the ability to detect associations. Finally, adjustment for variables such as income and occupation may partially account for pathways through which heat affects health and livelihoods, leading to more conservative estimates.

In contrast, perceived drought frequency was associated with increased odds of financial strain. This finding suggests that drought may affect households through economic and livelihood pathways, particularly in contexts where water availability and rainfed agriculture influences food production, market prices, or household expenditures. While extreme heat may be experienced primarily as immediate physical discomfort, drought may be more strongly linked to longer-term economic pressures, which may explain its stronger association with financial impacts in the present study.

Gender differences were observed in self-reported physical health impacts, with women having lower odds than men of reporting greater physical health impacts of extreme heat. This finding is different from the broader literature highlighted in the introduction section [13, 14], which suggests that women and girls often face heightened vulnerability to climate-related stressors due to gendered responsibilities such as water collection and food provision. However, the observed pattern in this study should be interpreted in the context of the study sample characteristics. The survey used a convenience sampling strategy and the sample predominantly consist of relatively young, highly educated respondents, many of whom reside in urban areas in South West Nigeria. About 60% of women in this sample are medium and high-income earners and 50% are professionals. They would therefore have different exposure patterns compared with women in rural area who are mostly low-income earners and with responsibilities such as water collection, agricultural labor, and other outdoor activities, which may increase exposure to extreme heat.

In urban environments, access to indoor spaces, electricity, fans, or other cooling resources may reduce direct exposure to heat. In contrast, men in the sample may have been more likely to engage in occupations or daily activities that require outdoor work or travel during the hottest parts of the day, potentially increasing their exposure to heat-related discomfort or illness. These findings highlight that gendered vulnerability to climate stressors is highly context-dependent and shaped by differences in occupation, mobility patterns, housing conditions, and access to resources. The findings from this study that underrepresents rural female population and individual engaged in climate-sensitive livelihoods such as agriculture, underscores the importance of examining climate impacts on gender within specific social and economic contexts.

Although some studies in Nigeria and across sub-Saharan Africa report no significant gender differences in health outcomes following weather shocks, climate events can affect men and women differently depending on labor roles, exposure patterns, and social norms [20]. For example, women’s agricultural labor may be reduced during droughts, while men may migrate in response to high temperatures, altering relative exposure to heat stress [20]. These context-specific differences likely contribute to the lower physical health impacts observed among women in our study and highlight the importance of considering gender in climate and health assessments.

The patterns of coping behaviors among study participants point to both strengths and gaps in adaptation at the household level. Participants reported a variety of behavioral adaptations, with the most common strategies combining indoor cooling actions (bathing, opening windows, using fans) with increased intake of water or cold drinks. The predominance of low-cost behaviors likely reflects both their feasibility and the limited availability of structural cooling options, such as air conditioning. These low-cost behaviors are consistent with public health recommendations and can be effective in moderating heat stress when implemented early and consistently. At the same time, very few participants reported more adaptive or community-oriented strategies, such as changing diets, checking on neighbors, or going somewhere other than home to stay cool. This suggests that coping is largely individualistic. The less frequent reporting of community-oriented strategies may indicate underutilized opportunities to leverage social cohesion, community education, and collective action as protective factors during periods of extreme heat [21]. Social capital may have a potential buffering role in mitigating climate-related stress ([22], and with respect to financial impacts, strong community networks may facilitate shared coping strategies, such as resource pooling during EWEs or emotional support during financial shocks.

The analysis of correlations between exposure and impacts highlights the complexity of heat-related risk pathways. In bivariate analyses, more frequent exposure to very hot temperatures was modestly associated with greater self-reported physical impacts, which is consistent with the biological plausibility of heat related illness and discomfort. In contrast, heat exposure was not significantly associated with mental health or financial impacts in the correlation analyses, while more frequent drought was strongly associated with reported financial strain. This pattern suggests that residents may perceive heat and drought through different lenses, with heat linked more directly to bodily sensations and immediate discomfort, and drought linked more directly to livelihoods, income, and longer-term financial security [23]. After accounting for gender, income, social cohesion, and geopolitical zone, perceived drought remained a strong predictor of financial impacts, with higher drought frequency associated with greater odds of reporting that finances were affected. This suggests that economic and livelihood pathways may be particularly sensitive to changes in water availability and agricultural or market conditions, and that these pathways may operate somewhat independently of the direct bodily experience of heat.

It is notable that income, neighborhood social cohesion, and geopolitical zone did not show strong associations with reported health and financial impacts. This could indicate that vulnerability to perceived heat-related and drought-related impacts is widespread across socioeconomic groups and regions in this setting. However, this should be interpreted with caution, as the sample consisted primarily of professionals from southwest Nigeria, which may underrepresent more vulnerable populations such as informal workers or rural farmers in northern zones. It could also be that the results reflect limited variability in these measures or sample size constraints that reduce statistical power to detect more modest associations. The weak association with social cohesion is particularly interesting given the very low prevalence of community-oriented coping behaviors. It may be that broader perceptions of cohesion do not automatically translate into concrete protective actions during environmental stress without targeted programs that activate and support those networks.

### 4.2. Measurement considerations

The study relies on self-reported impact of extreme heat and drought rather than objective exposure measurement. Exposure in this case may be influenced by ambient metrological conditions, housing characteristics, individual activities, and respondents in different climatic zones may interpret ‘very hot’ differently. This may introduce systematic perception bias in the findings.

Mental health impact was assessed with the question ‘To what extent is your [mental/physical] health negatively affected by very hot temperatures during the dry season?.’ This does not adequately interrogate mental health and limits sensitivity of the measurement. Cultural interpretations of psychological distress may further influence reporting. Therefore, lack of association between heat and mental health may be due to limitation of outcome measurement and study design.

### 4.3. Sampling and generalizability

The convenience sampling strategy, English-language eligibility and online distribution resulted in a sample that was predominantly young, highly educated, professional and concentrated in South West Nigeria. Populations most vulnerable to climate-related stressors, including rural agricultural workers, informal laborers, older adults and individuals with limited access to cooling and healthcare, are underrepresented. Consequently, prevalence estimates and socioeconomic gradients may be underestimated.

### 4.4. Policy and practice implications

The findings of this study support several practical directions that are directly grounded in the observed results.

First, households are already engaging in multiple low-cost individual coping behaviors, particularly hydration, bathing and the use of fans. Interventions should therefore prioritizes strengthening the safety and sustainability of these existing practices, including reliable access to safe drinking water and stable electricity to support basic cooling.

Second, the very low prevalence of community-oriented coping behaviors suggests missed opportunities for collective protection. Community-based education programs and locally coordinated support mechanisms could help translate social cohesion into practical protective actions during periods of extreme heat.

Third, the strong association between perceived drought and financial strain highlights the importance of integrating livelihood protection and income-stabilization measures into climate adaptation efforts, particularly for households whose income is sensitive to water availability.

Finally, the observed gender differences in physical health impacts indicate that adaptation strategies should be gender-responsive and sensitive to local labor patterns, mobility and access to resources.

### 4.5. Limitations and future research

A key limitation of this study relates to the measurement of extreme heat exposure. Participants were asked how often very hot temperatures occur during the dry season, which captures perceived weather conditions rather than individual-level heat exposure. Perceived weather reflects general ambient climatic conditions, whereas individual exposure depends on multiple factors, including occupation, time spent outdoors, housing characteristics, access to cooling resources, and personal coping strategies. As a result, two individuals experiencing the same ambient temperatures may have substantially different levels of actual heat exposure. Additionally, Nigeria spans several climatic zones, including the humid tropical monsoon climate in the southern coastal regions, the tropical wet-and-dry (savanna) climate in central areas, and semi-arid conditions in the northern regions. Therefore, the findings should not be interpreted as nationally representative.

Perceptions of extreme heat may therefore vary across regions due to differences in humidity, temperature, and seasonal weather patterns. Humidity, in particular, plays a significant role in shaping human heat perception and thermal discomfort. Consequently, respondents living in more humid southern regions may perceive temperatures as hotter than respondents in drier regions experiencing similar or even higher ambient temperatures. This climatic heterogeneity may introduce systematic bias when using self-reported measures of heat exposure. The present study did not incorporate objective meteorological data such as temperature, humidity, or drought indices due to the exploratory nature and funding limitations.

Future research could strengthen exposure assessment by integrating environmental data from ground-based meteorological stations or global datasets at regional or district levels. Combining self-reported survey with objective climate indicators would provide a more comprehensive understanding of how ambient environmental conditions translate into individual heat exposure and associated health and livelihood impacts.

More representative sampling approaches, including targeted recruitment of rural residents, informal workers and older adults, are essential to better characterize socioeconomic and regional inequalities in climate-related vulnerability. Longitudinal and mixed-methods designs would further improve understanding of how self-reported and objective exposure measures interact over time to influence health, wellbeing and livelihoods.

The use of a single question to measure mental health impact in this study limits the sensitivity of the measurement. Additionally, cultural differences in the understanding and expression of psychological distress may influence how participants interpret and respond to general questions about mental health. In some contexts, psychological symptoms may be underreported, expressed somatically, or not readily identified as mental health concerns. These factors may contribute to outcome misclassification and reduce the ability to detect meaningful associations.

The absence of significant associations between heat exposure and mental health outcomes should be interpreted with caution. The null findings may be due to limitations in measurement sensitivity, exposure and outcome misclassification. This is particularly important given a growing body of evidence linking heat exposure to psychological distress, sleep disruption, and exacerbation of pre-existing mental health conditions. Future research should incorporate validated and culturally appropriate mental health instruments to more accurately capture the psychological impacts of climate-related stressors.

Despite these limitations, the study offers valuable insight into how people experience and respond to extreme heat. It highlights the importance of addressing both immediate physical stress and longer-term financial strain, as well as to consider gendered patterns of vulnerability in climate and health policy.

## 5. Conclusion

This exploratory self-reported study shows that many Nigerian adults perceive extreme heat and drought as important stressors affecting physical health, mental wellbeing and household finances. While self-reported measure of extreme heat was not associated with adverse outcomes after adjustment, self-reported drought was strongly linked to financial strain. Gender differences in self-reported physical health impacts suggest that vulnerability patterns are context-specific and shaped by local exposure and social roles.

These findings highlight the need for future research that combines objective climate data with representative sampling and more detailed health measurement. In the interim, strengthening low-cost household coping practices, supporting community-based protective actions and integrating livelihood protection into climate adaptation strategies represent practical, evidence-aligned priorities for addressing climate-related stressors in Nigeria.

## Figures and Tables

**Figure A1.**
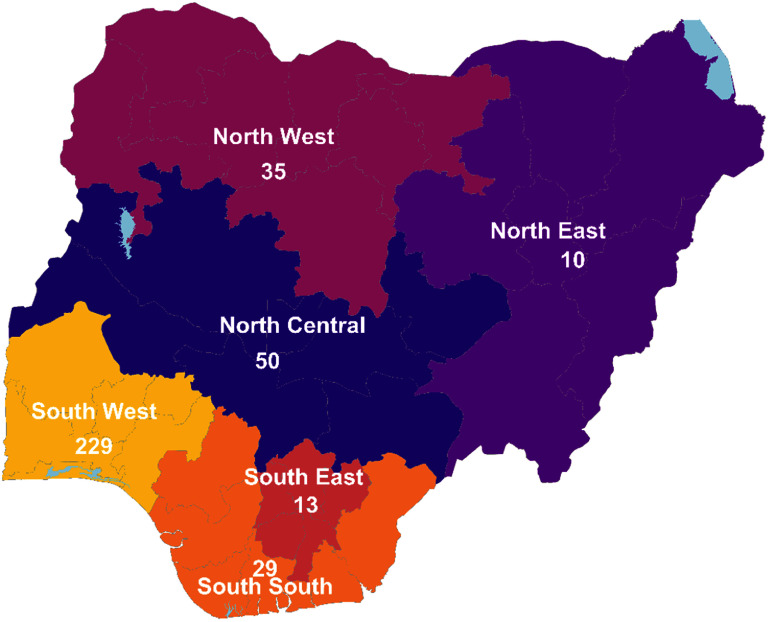
Number of participants by Nigerian geopolitical zone.

## Data Availability

All data that support the findings of this study are included within the article (and any supplementary information files).
